# Real‐World Effectiveness of Tolvaptan for Hyponatremia in Cirrhosis Across Global Regions: A Target Trial Emulation

**DOI:** 10.1002/jgh3.70395

**Published:** 2026-04-01

**Authors:** Kazuya Okushin, Ryosuke Tateishi, Mitsuhiro Fujishiro, Takeya Tsutsumi, Kazuhiko Koike, Tomohiro Tanaka

**Affiliations:** ^1^ Department of Gastroenterology Graduate School of Medicine, The University of Tokyo Tokyo Japan; ^2^ Department of Infection Control and Prevention Graduate School of Medicine, The University of Tokyo Tokyo Japan; ^3^ Kanto Central Hospital Tokyo Japan; ^4^ Division of Gastroenterology and Hepatology University of Iowa Carver College of Medicine Iowa USA; ^5^ Department of Health Management and Policy College of Public Health, University of Iowa Iowa USA

**Keywords:** ascites, hyponatremia, real world, target trial emulation, tolvaptan

## Abstract

**Background:**

Tolvaptan promotes electrolyte‐free water excretion and is widely used in Asia for ascites, but in Western countries its use is limited to hyponatremia because of hepatotoxicity concerns.

**Methods:**

We performed a retrospective cohort study using the TriNetX Research Network, emulating a target trial of cirrhotic patients with hyponatremia in global regions. Patients receiving tolvaptan plus sodium supplementation were compared with those receiving sodium supplementation alone. The primary outcome was 30‐day all‐cause mortality; secondary outcomes included correction of hyponatremia, changes in liver and renal function, and initiation of hemodialysis. Survival was analyzed using Kaplan–Meier curves with the log‐rank test and Cox proportional hazards models. Propensity score matching was applied to balance baseline covariates.

**Results:**

Among 188 707 eligible patients, 2130 received tolvaptan. After 1:1 matching, 1983 patients were analyzed in each group. Baseline characteristics were balanced, although serum sodium remained slightly lower in the tolvaptan group. Thirty‐day mortality was 14.4% in the tolvaptan group and 16.1% in the control group (HR 0.87, 95% CI 0.74–1.02; log‐rank *p* = 0.09). By the end of the follow‐up, serum sodium levels were significantly lower in the tolvaptan group. Changes in renal and liver function were comparable between groups, with no signal of excess hepatotoxicity. Rates of hemodialysis initiation did not differ significantly.

**Conclusions:**

In this large real‐world target trial emulation across global regions with ethnic diversity, in the context of longstanding safety concerns in cirrhosis, tolvaptan was not associated with excess short‐term mortality, with point estimates consistently favoring its use. Further studies are warranted to clarify its safety and effectiveness in diverse clinical settings.

**Abbreviations**
AASLD: American Association for the Study of Liver Diseases
ADPKD: autosomal dominant polycystic kidney disease
ALP: alkaline phosphatase
ALT: alanine aminotransferase
AST: aspartate aminotransferase
CI: confidence interval
EASL: European Association for the Study of the Liver
FDA: Food and Drug Administration
HCC: hepatocellular carcinoma
HIPAA: Health Insurance Portability and Accountability Act
HR: hazard ratio
INR: international normalized ratio
RCT: randomized controlled trial
STROBE: Strengthening the Reporting of Observational Studies in Epidemiology

## 1 Introduction

Ascites is one of the major complications of decompensated liver cirrhosis [1, 2]. Conventional management for ascites initiates with sodium restriction and natriuretic diuretics, whereas subsequent management strategies are in variety among guidelines [1, 2, 3, 4].

Tolvaptan, an oral vasopressin V2‐receptor antagonist, induces selective electrolyte‐free water excretion, reducing fluid overload without direct natriuresis. In patients with refractory ascites, a randomized trial demonstrated improved symptom control with tolvaptan compared to conventional diuretics alone [5]. Subsequent studies mainly from Japan have reported favorable short‐ and long‐term outcomes with tolvaptan in cirrhotic patients, without significant adverse effects on liver function [6, 7, 8, 9, 10].

However, the American Association for the Study of Liver Diseases (AASLD) and the European Association for the Study of the Liver (EASL) guidelines [1, 2] do not recommend the use of tolvaptan for patients with cirrhosis and ascites or volume overload. In the United States, due to concerns about hepatotoxicity (mainly hepatocellular/acute hepatitis pattern) [11], the Food and Drug Administration (FDA) limits its use to hospitalized patients with clinically significant hyponatremia and has removed the indication for use in patients with cirrhosis [12]. Furthermore, in the field of cardiology, tolvaptan is only considered for the treatment of heart failure with hyponatremia because of the lack of evidence for long‐term survival benefit [13, 14].

In this study, we applied a target trial emulation framework to evaluate the real‐world effectiveness and safety of tolvaptan in patients with cirrhosis who developed hyponatremia across globally diverse regions. We focused on two clinically relevant short‐term outcomes: 30‐day mortality and correction of hyponatremia. Specifically, we sought to determine whether tolvaptan use was associated with short‐term mortality or excess renal or hepatic adverse events in routine practice.

## 2 Methods

### 2.1 Study Design

We conducted a retrospective cohort study using data from the TriNetX Research Network, a federated health research platform that aggregates de‐identified electronic health records from 108 healthcare organizations across global regions, including Asia‐Pacific, Europe, Middle East, Africa, Latin America and the United States [15]. This platform enables real‐time, population‐level analyses while protecting patient privacy. The study was designed using a target trial emulation framework to replicate key elements of a randomized controlled trial, including eligibility criteria, treatment assignment, follow‐up, and analysis. Data captured included demographics, diagnoses, medications, procedures, and laboratory results. In accordance with the Health Insurance Portability and Accountability Act (HIPAA), all data were de‐identified. Due to the use of HIPAA‐compliant, de‐identified data and the determination that the study did not involve human subjects research, the University of Iowa Institutional Review Board waived the need of obtaining approval and informed consent. This study was conducted in accordance with the principles of the Declaration of Helsinki and was reported in compliance with the Strengthening the Reporting of Observational Studies in Epidemiology (STROBE) guidelines.

### 2.2 Eligibility Criteria

Eligible patients were adults aged ≥ 18 years at the index date with a diagnosis of cirrhosis, identified using validated ICD‐10 codes (K70.30, K70.31. K74.60, K74.69, K76.6) [16]. Patients were also required to have a diagnosis of hyponatremia (E87.1) within 14 days before the index date, and those with autosomal dominant polycystic kidney disease (ADPKD, ICD‐10 Q61.2, Q61.3) [17, 18] were excluded. Of note, TriNetX harmonizes data by mapping historical ICD‐9‐CM diagnoses prior to October 1, 2015, to ICD‐10‐CM using General Equivalence Mappings [19].

We identified patients starting January 1, 2014, shortly after the FDA Drug Safety Communication on April 30, 2013, which restricted tolvaptan use as noted elsewhere, to November 22, 2025. Two mutually exclusive cohorts were defined: patients who received tolvaptan plus sodium supplementation (tolvaptan group) and those who received sodium supplementation alone as a sham comparator and never received tolvaptan during the study period (control group). Sodium supplementation [1, 2], defined as any administration of sodium‐containing treatments (both intravenous and oral), regardless of intent to treat hyponatremia, was used as a pragmatic comparator to align index dates for hyponatremia management in both groups. The index date was the first date of tolvaptan administration (concurrent with sodium) for the tolvaptan group and the first date of sodium administration for the control group. Patients with any prior tolvaptan use before the index date were excluded. Once assigned to a cohort, patients were analyzed using an intention‐to‐treat approach, remaining in their original group regardless of subsequent treatment changes.

### 2.3 Study Outcomes and Follow‐Up

The primary outcome was 30‐day all‐cause mortality, and the secondary outcomes included serum sodium at the end of follow‐up and new initiation of hemodialysis within 30 days (identified using procedure codes such as CPT 90937 and ICD‐9 39.95), with patients who had received hemodialysis before the index date not classified as new initiators. Additional secondary outcomes included renal function parameters (serum sodium and creatinine) and liver function parameters (total bilirubin, ALT, AST, and INR), recorded using the most recent values available before the end of follow‐up. Follow‐up began on the index date and continued until the outcome occurred, death, loss to follow‐up, or 30 days, whichever came first. Given the short follow‐up period, death and hemodialysis were not treated as competing risks [20].

### 2.4 Propensity Score Matching

We used 1:1 nearest‐neighbor propensity score matching with a caliper of 0.2 standard deviations of the logit of the propensity score. The model included baseline demographics (age, sex, race, and ethnicity), cirrhosis etiology (alcoholic liver disease [ALD], metabolic dysfunction‐associated steatohepatitis, steatotic liver disease, hepatitis B with or without hepatitis D, hepatitis C, other chronic viral hepatitis), hepatic encephalopathy, detailed information on diuretic use, hepatocellular carcinoma (HCC), clinical variables (serum sodium, creatinine, bilirubin, albumin, aspartate aminotransferase [AST], alanine aminotransferase [ALT], alkaline phosphatase [ALP], and international normalized ratio [INR]), comorbidities such as hypertension, cardiovascular diseases, diabetes mellitus, acute and chronic kidney diseases, and syndrome of inappropriate secretion of antidiuretic hormone (SIADH), and treatment for hepatic encephalopathy and portal hypertension such as rifaximin, lactulose, and carvedilol. During the propensity‐score matching, continuous clinical variables are categorized into predefined ranges, with missing values constituting an additional category in the propensity score model. The complete list of ICD‐10 codes for the diagnoses used in the matching process is provided in Table S1.

### 2.5 Target Trial Emulation

We designed this study using a target trial emulation framework, in which the components of a hypothetical randomized controlled trial (RCT) were explicitly specified prior to analysis [21, 22]. In contrast to the conventional propensity score analyses, which typically adjust for confounding between treatment assignments, the target trial emulation framework begins by defining eligibility criteria, treatment strategies, assignment time (time zero), follow‐up period, outcomes, and causal contrasts of interest in advance.

In this study, we first defined the eligibility criteria (patients with cirrhosis and hyponatremia), treatment strategies (tolvaptan plus sodium supplementation vs. sodium supplementation alone), and the start of follow‐up (the date of treatment initiation). Patients were aligned at a clearly defined time zero to minimize immortal time bias. Baseline covariates were measured prior to treatment initiation to avoid adjustment for post‐exposure variables. Propensity score matching was subsequently applied within this predefined framework to approximate exchangeability between treatment groups. Detailed specifications of cohort definitions, variable coding, and outcome measures are provided in Table 1.

**TABLE 1:** Target trial emulation of comparisons between tolvaptan users and non‐users.

Protocol	Specification of target trials	Emulation of target trials
Eligibility criteria	– Adult patients aged ≥ 18 years – Had medical encounters with health care organizations between January 1, 2014, and November 22, 2025 – Had diagnoses of both cirrhosis and hyponatremia – Had tolvaptan plus sodium supplementation (tolvaptan group) or sodium supplementation alone as the treatment for hyponatremia – No history of autosomal dominant polycystic kidney disease	Same as for the target trials, except: – Each patient's index date was defined as the date of first prescription of either tolvaptan or sodium supplementation during the study period (January 1, 2014, through November 22, 2025).
Treatment strategies	Main analysis For the target trial comparing tolvaptan plus sodium supplementation and sodium supplementation alone – First prescription of tolvaptan – First prescription of sodium supplementation	Same as for the target trials.
		Patients in each cohort were required to have at least one treatment session.
Treatment assignment	Individuals are randomly assigned to one of the comparators at baseline.	Individuals are assigned to the comparator according to the record of their medication use and assumed randomization by propensity‐score matching for the covariates.
Outcomes	Primary outcome – 30‐day all‐cause mortality	Same as for the target trials
	Secondary outcomes – New initiation of hemodialysis within 30 days – Renal and liver function parameters	
Follow‐up	Follow‐up for each individual will start at treatment assignment and end on day of death (primary outcome), end of study, or loss to follow‐up, whichever occurs first.	Same as for the target trials
Casual contrast of interest	Intention‐to‐treat	Observational analog to intention‐to‐treat
Statistical analysis	– Kaplan–Meier curve to assess cumulative incidences of the comparator during follow‐up period. – Cox proportional hazards regression to compare the risk of the preplanned outcomes on a daily basis during the follow‐up period. – Subgroup analysis to assess the overall survival across prespecified patient populations stratified by demographics, disease characteristics, and treatment regimen.	Same as for the target trial except observational analogs of intention‐to‐treat analyses required matching for confounding variables by propensity‐score matching.

### 2.6 Statistical Analysis

Baseline characteristics were summarized as means with standard deviations for continuous variables and counts with percentages for categorical variables. Kaplan–Meier survival curves were generated to compare outcomes, with differences assessed by the log‐rank test. Cox proportional hazards models were used to estimate hazard ratios (HRs) and 95% confidence intervals (CIs). Analyses were performed univariately, based on the assumption that key measurable confounders were adequately balanced through propensity score matching. A two‐sided *p*‐value < 0.05 was considered statistically significant. All analyses were conducted within the TriNetX platform, and Kaplan–Meier survival curves were described using R software version 4.5.1 (R Foundation, Vienna, Austria, http://www.r‐project.org/).

## 3 Results

### 3.1 Patient Characteristics

A total of 188 707 patients met eligibility criteria, including 2130 patients in the tolvaptan group and 176 577 patients in the control group. After 1:1 propensity score matching, 1983 patients remained in each cohort. Baseline characteristics were well balanced across most variables (Table 2). Before matching, the tolvaptan group had lower mean serum sodium (124.1 ± 5.2 mmol/L vs. 132.5 ± 5.6 mmol/L), higher prevalence of kidney diseases (59.3% vs. 24.3%), and frequent usage of medication for hepatic encephalopathy and portal hypertension, whereas the prevalence of HCC was similar between the groups. After matching, the groups were well balanced, although sodium remained slightly lower in the tolvaptan group (124.2 ± 5.3 mmol/L vs. 127.2 ± 6.2 mmol/L; SMD = 0.522). Notably, serum total bilirubin and albumin, key components of the ALBI score reflecting liver functional reserve [23], showed imbalance before matching but achieved adequate balance after matching (Supplementary Figure 1).

**TABLE 2:** Background characteristics of patients treated with or without tolvaptan.

Characteristics	Before PSM			After PSM		
	Tolvaptan (*n* = 2130)	Sodium alone (*n* = 176 577)	SMD	Tolvaptan (*n* = 1983)	Sodium alone (*n* = 1983)	SMD
Demographics						
Age, mean (SD)	61.5 (12.6)	58.5 (13.0)	0.231	61.4 (12.7)	61.3 (11.9)	0.013
Male, *n* (%)	1333 (62.6)	108 208 (61.3)	0.027	1244 (62.7)	1249 (63.0)	0.005
Race, *n* (%)						
White	1699 (79.8)	130 751 (74.0)	0.136	1576 (79.5)	1589 (80.1)	0.016
Black/African American	179 (8.4)	19 932 (11.3)	0.097	171 (8.6)	166 (8.4)	0.009
Asian	84 (3.9)	5740 (3.3)	0.037	77 (3.9)	67 (3.4)	0.027
Other Race	66 (3.1)	8298 (4.7)	0.083	63 (3.2)	59 (3.0)	0.012
Unknown Race	81 (3.8)	8147 (4.6)	0.040	76 (3.8)	82 (4.1)	0.015
Ethnicity, *n* (%)						
Hispanic or Latino	219 (10.3)	18 175 (10.3)	< 0.001	197 (9.9)	186 (9.4)	0.019
Not Hispanic or Latino	1467 (68.9)	130 931 (74.1)	0.117	1364 (68.8)	1375 (69.3)	0.012
Unknown Ethnicity	444 (20.8)	27 471 (15.6)	0.137	422 (21.3)	422 (21.3)	< 0.001
Diagnosis						
Etiology, *n* (%)						
Alcoholic liver disease	628 (29.5)	30 228 (17.1)	0.296	597 (30.1)	638 (32.2)	0.045
Metabolic dysfunction‐associated steatohepatitis	152 (7.1)	8572 (4.9)	0.096	143 (7.2)	159 (8.0)	0.030
Fatty (change of) liver, not elsewhere classified	164 (7.7)	7660 (4.3)	0.142	150 (7.6)	173 (8.7)	0.042
Chronic viral hepatitis	141 (6.6)	9231 (5.2)	0.059	137 (6.9)	144 (7.3)	0.014
Chronic viral hepatitis C	116 (5.4)	7842 (4.4)	0.046	112 (5.6)	117 (5.9)	0.011
Chronic viral hepatitis B without delta‐agent	26 (1.2)	1564 (0.9)	0.033	26 (1.3)	25 (1.3)	0.004
Chronic viral hepatitis B with delta‐agent	10 (0.5)	43 (0.0)	0.090	10 (0.5)	10 (0.5)	< 0.001
Chronic viral hepatitis, unspecified	10 (0.5)	47 (0.0)	0.089	10 (0.5)	10 (0.5)	< 0.001
Hepatic encephalopathy	208 (9.8)	9333 (5.3)	0.170	201 (10.1)	216 (10.9)	0.025
Hepatocellular carcinoma	82 (3.8)	8302 (4.7)	0.042	77 (3.9)	79 (4.0)	0.005
Hypertensive diseases	1525 (71.6)	56 414 (31.9)	0.864	1402 (70.7)	1426 (71.9)	0.027
Other heart disease including heart failure	1355 (63.6)	40 882 (23.2)	0.894	1249 (63.0)	1327 (66.9)	0.083
Ischemic heart diseases	802 (37.7)	21 436 (12.1)	0.617	736 (37.1)	758 (38.2)	0.023
Venous diseases including portal vein thrombosis	574 (26.9)	26 776 (15.2)	0.292	532 (26.8)	569 (28.7)	0.042
Other disorders of the circulatory system	608 (28.5)	15 642 (8.9)	0.522	562 (28.3)	579 (29.2)	0.019
Peripheral arterial disease	321 (15.1)	10 189 (5.8)	0.308	289 (14.6)	307 (15.5)	0.025
Pulmonary heart disease and diseases of pulmonary circulation	468 (22.0)	10 366 (5.9)	0.478	431 (21.7)	460 (23.2)	0.035
Chronic rheumatic heart diseases	329 (15.4)	5381 (3.0)	0.438	291 (14.7)	276 (13.9)	0.022
Diabetes mellitus	792 (37.2)	36 247 (20.5)	0.374	733 (37.0)	757 (38.2)	0.025
Malnutrition	564 (26.5)	15 236 (8.6)	0.483	503 (25.4)	528 (26.6)	0.029
Cerebrovascular diseases	240 (11.3)	6646 (3.8)	0.288	216 (10.9)	224 (11.3)	0.013
Acute kidney failure and chronic kidney disease	1264 (59.3)	42 851 (24.3)	0.761	1170 (59.0)	1224 (61.7)	0.056
Chronic lower respiratory diseases	615 (28.9)	20 214 (11.4)	0.445	565 (28.5)	591 (29.8)	0.029
SIADH	394 (18.5)	1336 (0.8)	0.631	324 (16.3)	300 (15.1)	0.033
Medication						
Rifaximin	316 (14.8)	12 556 (7.1)	0.249	296 (14.9)	323 (16.3)	0.038
Lactulose	606 (28.5)	22 443 (12.7)	0.397	567 (28.6)	618 (31.2)	0.056
Nadolol	24 (1.1)	2392 (1.4)	0.021	23 (1.2)	27 (1.4)	0.018
Carvedilol	267 (12.5)	7492 (4.2)	0.303	246 (12.4)	249 (12.6)	0.005
Propranolol	74 (3.5)	3567 (2.0)	0.089	66 (3.3)	73 (3.7)	0.019
Spironolactone	789 (37.0)	24 234 (13.7)	0.556	730 (36.8)	785 (39.6)	0.057
Eplerenone	26 (1.2)	355 (0.2)	0.122	23 (1.2)	18 (0.9)	0.025
Amiloride	19 (0.9)	444 (0.3)	0.085	17 (0.9)	24 (1.2)	0.035
Furosemide	1328 (62.3)	35 609 (20.2)	0.948	1221 (61.6)	1311 (66.1)	0.095
Bumetanide	317 (14.9)	3986 (2.3)	0.463	280 (14.1)	282 (14.2)	0.003
Torsemide	198 (9.3)	3210 (1.8)	0.331	181 (9.1)	176 (8.9)	0.009
Laboratory values, mean (SD)						
Sodium (mmol/L)	124.1 (5.2)	132.5 (5.6)	1.568	124.2 (5.3)	127.2 (6.2)	0.522
Available, *n* (%)	2010 (94.4)	91 171 (51.6)		1863 (93.9)	1886 (95.1)	
Creatinine (mg/dL)	1.3 (0.9)	1.6 (3.4)	0.140	1.3 (1.0)	1.3 (1.0)	0.066
Available, *n* (%)	1949 (91.5)	88 940 (50.4)		1806 (91.1)	1838 (92.7)	
Total bilirubin (mg/dL)	3.0 (4.9)	4.0 (6.7)	0.171	3.1 (5.0)	3.5 (5.7)	0.071
Available, *n* (%)	1895 (89.0)	81 700 (46.3)		1750 (88.3)	1807 (91.1)	
Albumin (g/dL)	3.0 (0.7)	3.1 (0.7)	0.093	3.0 (0.7)	3.0 (0.7)	0.015
Available, *n* (%)	1927 (90.5)	80 588 (45.6)		1781 (89.8)	1787 (90.1)	
International normalized ratio	1.6 (0.7)	1.6 (0.7)	0.029	1.6 (0.7)	1.7 (0.7)	0.041
Available, *n* (%)	1587 (74.5)	62 180 (35.2)		1466 (73.9)	1524 (76.9)	
Alanine aminotransferase (U/L)	45.6 (113.4)	54.7 (154.8)	0.067	46.3 (117.3)	49.3 (129.3)	0.024
Available, *n* (%)	1933 (90.8)	82 926 (47.0)		1787 (90.1)	1836 (92.6)	
Aspartate aminotransferase (U/L)	69.0 (126.8)	89.9 (266.7)	0.100	69.9 (130.8)	77.3 (165.9)	0.050
Available, *n* (%)	1928 (90.5)	82 020 (46.4)		1785 (90.0)	1831 (92.3)	
Alkaline phosphatase (U/L)	162.5 (171.0)	177.0 (176.5)	0.083	162.8 (172.6)	161.4 (157.7)	0.008
Available, *n* (%)	1920 (90.1)	81 657 (46.2)		1775 (89.5)	1826 (92.1)	

*Note:* Laboratory variables are summarized among patients with available measurements. For each laboratory variable, the number and proportion of patients with available data are shown before and after propensity score matching.

Abbreviations: SD, standard deviation; SIADH, syndrome of inappropriate secretion of antidiuretic hormone; SMD, standardized mean difference.

### 3.2 Short‐Term Mortality

**FIGURE 1:**
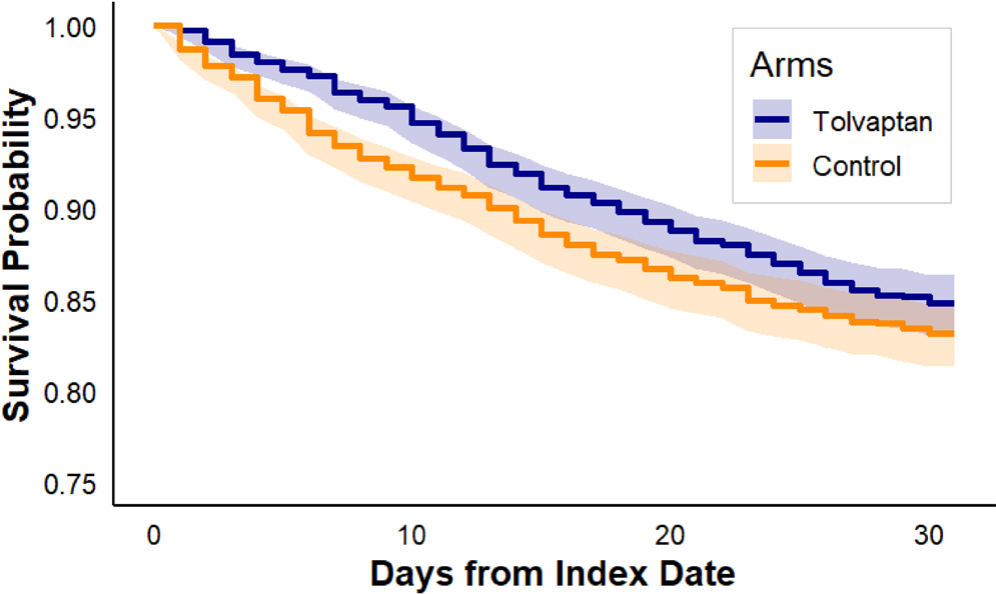
Kaplan–Meier Curve for 30‐Day Mortality for patients treated with or without tolvaptan. Baseline matched cohorts: *N* = 1983 in each group; 30‐day deaths: 286 (tolvaptan) versus 320 (control). Time‐specific numbers at risk are not provided by TriNetX.

At 30 days, mortality occurred in 286 of 1983 patients (14.4%) in the tolvaptan group versus 320 of 1983 patients (16.1%) in the control group (log‐rank *p* = 0.09). The Kaplan–Meier survival curve is shown in Figure 1. The hazard ratio was 0.87 (95% CI, 0.74–1.02), suggesting a directionally favorable but statistically non‐significant association with 30‐day survival.

### 3.3 Secondary Outcomes

**FIGURE 2:**
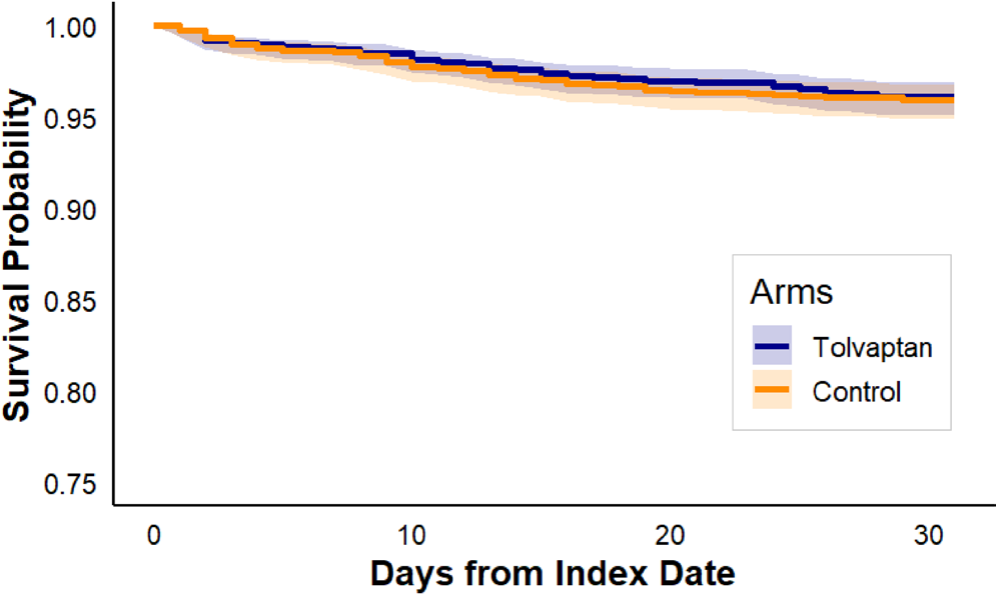
Kaplan–Meier Curve for 30‐Day Dialysis Initiation for patients treated with or without tolvaptan. Baseline matched cohorts: *N* = 1895 (tolvaptan) and *N* = 1906 (control); 30‐day hemodialysis initiation: 68 (3.6%) versus 71 (3.7%). Time‐specific numbers at risk are not provided by the TriNetX.

By the end of the follow‐up, serum sodium levels were significantly lower in the tolvaptan group compared with the control group (131.7 ± 133.1 mmol/L, *p* < 0.001). The need for initiating hemodialysis during the 30‐day follow‐up was similar between groups, with 68 of 1895 patients (3.6%) in the tolvaptan group and 71 of 1906 patients (3.7%) in the control group (log‐rank *p* = 0.76, Figure 2). The hazard ratio was also non‐significant (HR = 0.95, 95% CI: 0.68–1.33). Numbers of abdominal paracenteses were similar between the groups (Table 3).

**TABLE 3:** Outcome majors of patients with cirrhosis who developed hyponatremia at the end of the follow‐up.

Characteristics	Tolvaptan (*n* = 1983)	Sodium alone (*n* = 1983)	*p*
Procedure, mean (SD)			
Number of abdominal paracenteses	0.36 (1.02)	0.33 (0.93)	0.45
Laboratory values, mean (SD)			
Sodium (mmol/L)	131.7 (5.8)	133.1 (5.7)	< 0.001
Creatinine (mg/dL)	1.4 (1.1)	1.4 (1.2)	0.28
Total bilirubin (mg/dL)	3.4 (5.9)	3.5 (6.0)	0.55
International normalized ratio	1.8 (0.9)	1.7 (0.8)	0.42
Alanine aminotransferase (U/L)	46.5 (95.9)	52.3 (190.9)	0.29
Aspartate aminotransferase (U/L)	76.7 (194.3)	89.1 (352.8)	0.23

Abbreviation: SD, standard deviation.

Creatinine levels were not significantly difference between the groups. By the end of follow‐up, liver‐related parameters showed no significant differences whereas, notably, both ALT and AST levels were relatively lower in the tolvaptan group compared with the control group (46.5 ± 95.9 U/L vs. 52.3 ± 190.9 U/L, *p* = 0.29 for ALT; 76.7 ± 194.3 U/L vs. 89.1 ± 352.8 U/L, *p* = 0.23 for AST).

## 4 Discussion

In this real‐world target trial emulation, tolvaptan was not significantly associated with 30‐day mortality in cirrhotic patients with hyponatremia despite persistent safety concerns in this setting, with point estimates across multiple analytical approaches consistently favoring tolvaptan. The marginal survival benefit was observed without an increased risk of hemodialysis initiation, with numerically lower creatinine levels by day‐30, and liver function parameters remained stable. Although correction of hyponatremia, which could not be standardized by the propensity score matching at the baseline, was not enough, these findings suggest that tolvaptan, when administered in routine clinical practice, may offer a short‐term prognostic benefit to a population for whom therapeutic options are limited.

While our findings do not demonstrate a statistically significant survival benefit, the direction of effect is broadly consistent with several prior observational studies from Japan reporting favorable short‐ and long‐term outcomes among tolvaptan users [6, 7, 8, 9, 10]. Although we only analyzed short‐term prognosis, reflecting regulatory restrictions in some regions, particularly in the United States where tolvaptan use is generally limited to short‐term treatment (up to 30 days) [24], long‐term treatment experiences and prognostic evidence have been accumulated mainly in Japan [7, 8, 9, 10]. In this context, the present analysis provides complementary evidence by examining short‐term outcomes in a large, ethnically diverse cohort across global regions.

Besides the controversial survival benefits, U.S. and European guidelines have not endorsed tolvaptan use in cirrhosis, primarily due to concerns about hepatotoxicity [11]. However, in this study, no clear sign of excess liver injury was observed in the tolvaptan group during the 30‐day follow‐up. Liver injury has also been infrequently reported in Japanese studies, including a clinical trial [5]. A prior dose‐finding study identified 7.5 mg/day as the optimal dose and found that 30 mg/day was associated with higher rate of hyperbilirubinemia (12.0%) compared with 7.5 mg/day and 15 mg/day (both 4.0%) [25]. Consequently, tolvaptan is initiated at 3.75 mg/day and titrated up to 7.5 mg/day is used for cirrhosis in treating ascites with or without hyponatremia in Japan, whereas in the United States, 15–60 mg/day is approved for hyponatremia and 60–120 mg/day is approved for autosomal dominant polycystic kidney disease (ADPKD) [24]. Taken together, these findings suggest that lower doses of tolvaptan which is recommended in the guidelines by the Japan Society of Gastroenterology and the Japan Society of Hepatology [3, 4], may be safer for patients with cirrhosis. Our cohort included only patients treated since January 2014, following the FDA safety warning issued in April 2013 [12], which may have let to closer monitoring for liver injury and potential adjustment in dosing. Given the observational nature of the data and the limited follow‐up duration, these findings should not be interpreted as definitive evidence of hepatic safety, but rather as supportive short‐term observations that warrant further investigation, particularly with respect to dosing strategies and longer‐term exposure.

The study also provides important insight into renal outcomes. Compared to the controls, tolvaptan did not significantly increase the incidence of new hemodialysis initiation or worsen renal function. The lower mortality rate suggests that volume management may have provided a clinical benefit. This finding is clinically significant because renal dysfunction remains a key factor in determining the prognosis of advanced cirrhosis [26].

Several limitations inherent to this observational target trial emulation warrant consideration. First, although we carefully specified eligibility criteria, treatment strategies, time zero, and follow‐up in accordance with the target trial framework, residual confounding cannot be fully excluded. Propensity score matching balanced measured baseline covariates; however, residual confounding inherent to observational data cannot be fully excluded. Second, the TriNetX platform does not provide access to certain granular clinical variables. In particular, imaging data reflecting the severity of ascites and individual‐level Child‐Pugh or MELD‐Na scores were unavailable; therefore, adjustment for baseline liver functional reserve may have remained incomplete. Nevertheless, we incorporated and balanced available variables related to these scores, including relevant laboratory values, ICD‐10 codes for ascites and hepatic encephalopathy, and treatment‐related factors such as diuretics, lactulose and rifaximin. Third, serum sodium levels remained slightly lower in the tolvaptan group both at baseline after matching and at 30 days. This likely reflects preferential use of tolvaptan in patients with more severe or refractory hyponatremia. Nevertheless, mortality tended to be lower in the tolvaptan group despite lower baseline sodium levels, supporting the robustness of the observed association. Fourth, detailed information on treatment dosage and indication for both tolvaptan and sodium supplementation was not available due to the nature of the database. Consequently, sodium supplementation could not be characterized by dose or clinical intent, and regional differences in indications for tolvaptan could not be examined. Accordingly, further studies incorporating indication‐ and dose‐specific information are warranted to better define the role of tolvaptan in this population. Finally, follow‐up was limited to 30 days in our study, and evaluation of long‐term safety and survival outcomes should be needed in future studies.

In conclusion, although substantial safety concerns have surrounded its use, this target trial emulation did not demonstrate a statistically significant association between tolvaptan use and short‐term mortality in cirrhotic patients with hyponatremia. The consistent direction of effect across multiple analyses suggests that the role of tolvaptan in this population warrants further evaluation, including in regions outside Asia.

## Funding

This work was supported by UTokyo Global Activity Support Program for Young Researchers (K.O.), K08HS029195‐01A from the Agency for Healthcare Research and Quality (T.T.), and Health, Labour, and Welfare Policy Research Grants from the Ministry of Health, Labour, and Welfare of Japan (Policy Research for Hepatitis Measures [23HC2001], K.K.).

## Conflicts of Interest

The authors declare no conflicts of interest.

## Supporting information


**Supplementary Figure 1.** Supplementary Figure.

**Supplementary Table 1.** ICD‐10 codes for case definition.

## Data Availability

The datasets generated during the current study are not publicly available but can be obtained from the corresponding author upon reasonable request.
