# Supplementary figures and images for: Real‐World Effectiveness of Tolvaptan for Hyponatremia in Cirrhosis Across Global Regions: A Target Trial Emulation
Source: JGH Open. 2026 Apr 1;10(4):e70395. doi: 10.1002/jgh3.70395 (PMC13045325; doi:10.1002/jgh3.70395)

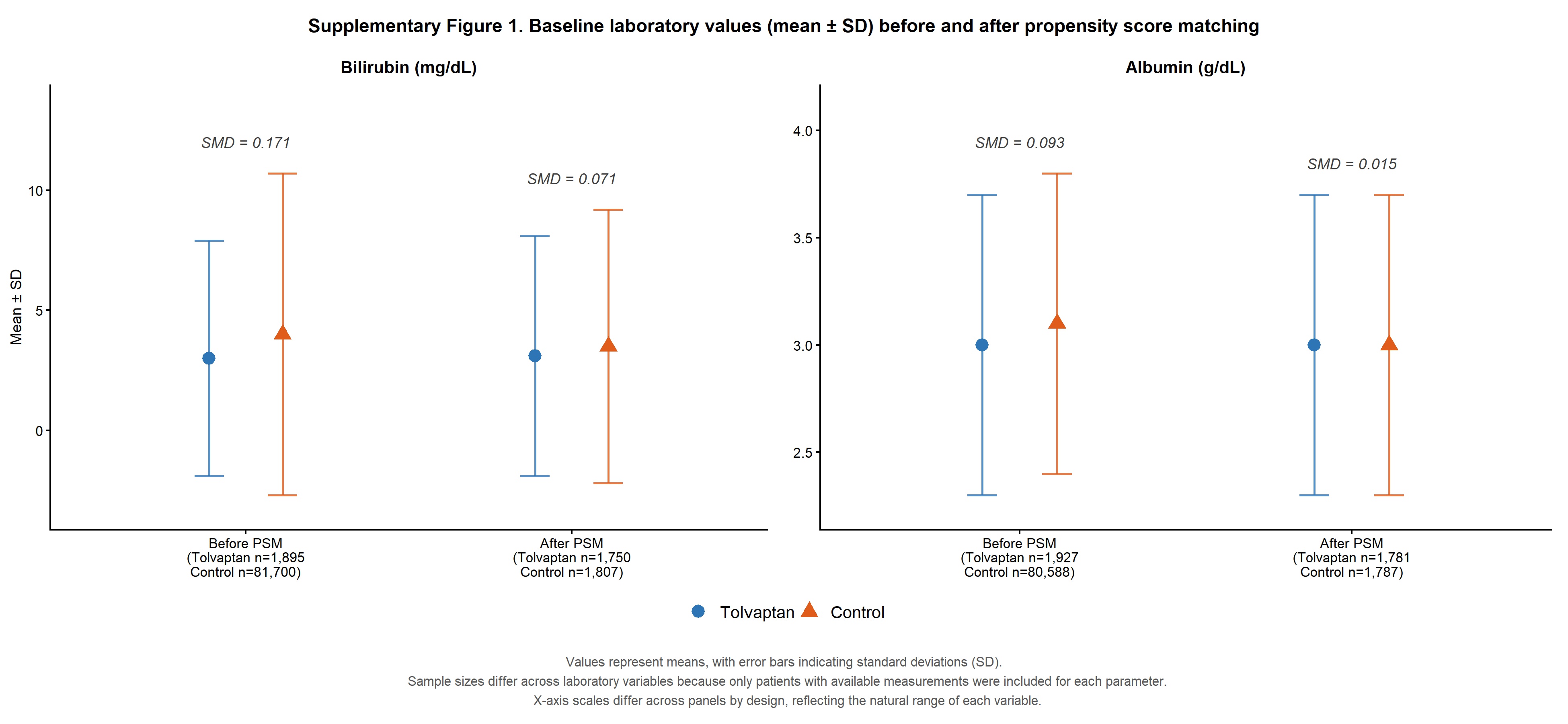

Supplement: Supplementary file 1 — Supplementary Figure 1. Supplementary Figure. [file JGH3-10-e70395-s001.jpg]
