# Supplementary material for: Real‐World Effectiveness of Tolvaptan for Hyponatremia in Cirrhosis Across Global Regions: A Target Trial Emulation
Source: JGH Open. 2026 Apr 1;10(4):e70395. doi: 10.1002/jgh3.70395 (PMC13045325; doi:10.1002/jgh3.70395)
Supplement: Supplementary file 2 — Supplementary Table 1. ICD‐10 codes for case definition. [file JGH3-10-e70395-s002.docx]

# Supplementary Table 1. ICD-10 codes for case definition.

| **Diagnosis** | **ICD-10 code** |
| --- | --- |
| Cirrhosis | K70.30, K70.31. K74.60, K74.69, K76.6 |
| Hyponatremia | E87.1 |
| Autosomal dominant polycystic kidney disease | Q61.2, Q61.3 |
| Alcoholic liver disease | K70 |
| Metabolic dysfunction-associated steatohepatitis | K75.81 |
| Steatotic liver disease | K76.0 |
| Hepatitis B | B18.0, B18.1 |
| Hepatitis C | B18.2 |
| Other chronic viral hepatitis | B18, B18.8, B18.9 |
| Hepatic encephalopathy | K76.82 |
| Hepatocellular carcinoma | C22.0 |
| Hypertensive diseases | I10-I1A |
| Other heart disease including heart failure | I30-I5A |
| Ischemic heart diseases | I20-I25 |
| Venous disease including portal vein thrombosis | I80-I89 |
| Other disorders of the circulatory system | I95-I99 |
| Peripheral arterial disease | I70-I79 |
| Pulmonary heart disease and diseases of pulmonary circulation | I26-I28 |
| Chronic rheumatic heart diseases | I05-I09 |
| Diabetes mellitus | E08-E13 |
| Malnutrition | E40-E46 |
| Cerebrovascular diseases | I60-I69 |
| Acute kidney failure and chronic kidney disease | N17-N19 |
| Chronic lower respiratory diseases | J40-J4A |
| Syndrome of inappropriate secretion of antidiuretic hormone | E22.2 |
